# Internal Qualification and Credentialing of Radiation Oncology Physicists to Perform Patient Special Procedures

**DOI:** 10.3389/fonc.2013.00319

**Published:** 2014-01-02

**Authors:** Michael D. Mills

**Affiliations:** ^1^Department of Radiation Oncology, University of Louisville School of Medicine, Louisville, KY, USA

**Keywords:** safety, special procedures, radiation oncology, medical physics, continuing education credits

## Abstract

In the arena of radiation oncology special procedures, medical physicists are often the focus professionals for implementation and administration of advanced and complex technologies. One of the most vexing and challenging aspects of managing complexity concerns the ongoing internal qualification and credentialing of radiation oncology physicists to perform patient special procedures. To demonstrate ongoing qualification, a physicist must: (a) document initial training and successful completion of competencies to implement and perform this procedure, (b) demonstrate familiarity with all aspects of the commissioning and quality assurance process, (c) demonstrate continuing education respecting this procedure, (d) demonstrate the peer-reviewed completion of a minimum number of patient special procedures during a specified time span, and (e) demonstrate satisfactory overall progress toward maintenance of specialty board certification. In many respects, this information complement is similar to that required by an accredited residency program in therapy physics. In this investigation, we report on the design of a management tool to qualify staff radiation oncology physicists to deliver patient procedures.

## Introduction

Consider the following hypothetical scenario: You are the director of an intermediate-sized academic center. Your institution treats 1500 external beam and 250 brachytherapy patients per year; about 175 of these are high dose rate afterloading (HDR) procedures. You are at your desk one morning leisurely reading a recently published task group report when a call comes in with a problem. Your primary HDR physicist is a nationally respected authority, but he is on vacation and out of the country. Your first backup physicist is on emergency medical leave tending to a family member in another state recovering from surgery. Your third backup called in sick this morning with flu symptoms. Your final emergency backup is in Chicago, attending the annual meeting of the Radiological Society of North America. The patient was scheduled for six routine Tandem and Ovoid procedures (T&O) and this is treatment number 4. You have not performed this procedure in over 5 years, but at one time you were the primary HDR physicist and have performed hundreds of treatment deliveries. The only other qualified medical physicist (QMP) in the clinic today who has ever treated an HDR procedure has not performed one in over a year, and that was on equipment from another vendor. There is one physics resident present who has recently checked off this procedure in his competency list. The radiation therapist who operates the console is present. You have a solid quality assurance program with multiple checklists for both the planning and delivery process. All certified medical physicists are qualified on your radioactive materials license to perform all brachytherapy procedures, including HDR. Do you, the physicist with marginal experience and the resident treat the patient? On what basis do you treat?

There is little guidance to be found in the literature to answer questions that deal with local credentialing. Yet this is a question every physics program must answer. What constitutes a local credentialing program? Who is qualified to perform a special procedure? How is qualification attained? How is it maintained? What happens if a medical physicist loses such qualification through necessity or neglect? A modern radiation oncology program has dozens of special procedures. How is it possible to keep up with which QMP is qualified to do what? Is the effort to do this necessary and cost effective? The purpose of this article is to develop an approach to answer these questions and provide some guidance in developing such a program.

## Methods

A physics director for a center of significant size might want to record these work components as part of an overall quality assurance program:

- The primary, secondary, tertiary, and emergency responsibility of each clinical staff member
- Radioactive materials license listing of individuals as qualified expert medical physicists as applicable
- The specific special procedures which require local privileges
- The number of patient procedures that must be performed under supervision within a specific time frame to qualify a professional for privileges
- The number of patient procedures that must be performed under supervision within a specific time frame to reinstate a professional for privileges after losing them for any reason
- The number of patient procedures that must be performed within a specific time frame to maintain privileges
- The number of continuing education hours specific to this special procedure that must be completed within a time frame to maintain privileges for a special procedure
- The training responsibilities and productivity training others for specific special procedures

These are some of the same types of questions that a program director for a CAMPEP accredited therapy physics residency program must face. These questions should be answered in some detail and recorded to withstand periodic peer review. Zacarias and Mills (1) addressed some of the components of appropriate recording for a CAMPEP accredited residency program.

The medical physicists with primary, secondary, and tertiary responsibilities should be in regular rotation for planning, validation, and delivery responsibilities. The medical physicist with emergency responsibilities may not be in regular rotation, but must complete a certain number of cases to include all planning, validation, and delivery activities within a reasonable time period. This time period may vary depending on the number and frequency of cases available, the complexity of the procedure, and the opportunities for requalification if initial privileges are lost. Of course, having four levels of support is a luxury in many institutions. Nevertheless, the same logic may be applied with fewer personnel, just for fewer levels of support. Additionally, some procedures that may be scheduled with flexibility are so uncommon that only one or two physicists maintain clinical competence. This is acceptable provided the entire clinical team is comfortable with the administrative details of the overall quality assurance program. Note that training is an important component to maintain local privileges. Attending or teaching seminars, literature reviews such as a “journal club,” physics peer review of special procedure cases, and training of physician and physics residents are all components of ongoing training to maintain quality. There are vanishingly few resources that deal with establishing and maintaining competency in specific radiation oncology special procedures. One notable exception is Solberg et al. (2).

## Results

The first logical step in implementing this program is to decide what special procedures require records and to decide what records to record. It is desirable to keep such recording to the minimum required to maintain the integrity of an excellent program as many radiation oncology professionals already work long hours. Table 1 illustrates conceptually the minimum components to design a local credentialing quality assurance program. Note that the recorded information includes the number of cases to be completed within a given time frame. The example shown is quarterly, but you may find it necessary to require monthly reporting for some special procedures and annual reporting for others, depending on the frequency. If a physicist fails to achieve the specified quota for any reason, the competency may be restored by performing a number of cases under supervision during a specified time period and perhaps completing targeted continuing education activities.

**Table 1:** **The special procedure records required for a medical physicist to establish and maintain clinical competency are listed**.

Special procedure (S.P.) broad category	Number of cases completed under supervision/time frame required to establish competency	Number of cases completed/time frame required to maintain or restore competency	Number of S.P. continuing education hours/time frame required to establish/maintain competency	Expected number of individuals to be trained/time frame to maintain status as domain expert in this special procedure
Total skin irradiation	4/quarter	2/quarter	4/2/year	1/year
Total body irradiation	4/quarter	2/quarter	4/2/year	1/year
Stereotactic body radiotherapy	10/quarter	5/quarter	6/4/year	1/year
Stereotactic radiosurgery	10/quarter	5/quarter	6/4/year	1/year
Intraoperative radiotherapy	4/quarter	2/quarter	4/2/year	1/year
Low dose rate brachytherapy	4/quarter	2/quarter	4/2/year	1/year
Prostate seed brachytherapy	4/quarter	2/quarter	4/2/year	1/year
High dose rate brachytherapy	6/quarter	3/quarter	6/4/year	1/year
Therapeutic nuclear medicine	4/quarter	2/quarter	4/2/year	1/year
CT/PET/MRI fusion	4/quarter	2/quarter	4/2/year	1/year
4D respiratory gating	6/quarter	3/quarter	6/4/year	1/year
Gamma Knife radiosurgery	10/quarter	5/quarter	6/4/year	1/year
CyberKnife radiosurgery	10/quarter	5/quarter	6/4/year	1/year
TomoTherapy IGRT	6/quarter	3/quarter	6/4/year	1/year

*Numbers presented are for example only and will not necessarily represent the numbers you might select for your clinic*.

It is expected that any staff medical physicist involved in the commissioning and clinical enactment of new technology should undergo thorough training by the manufacturer. The number of hours and overall scope of training will vary by the type and complexity of the technology being implemented. For the technologies listed, 2 days up to 1 week of initial training are common.

The medical physicist must complete and document a certain number of continuing education hours dealing directly with the special procedure to maintain credentialing. Finally, the staff member may be expected to train a minimum number of individuals over a year if the institution includes a clinical training program of some type. This latter requirement may be optional, depending on what training programs are offered in the institution.

The next step in this administrative formalism is to determine a method for capturing this information, recording it, and reporting it. There are two logical options. Either have the QMP report the activities in a spreadsheet or report form or have the QMP report both the activities and record a matrix demonstrating that competency is maintained. If the program is not too large, it may be more desirable to use the former option and leave the determination of competency to the administrative director. The required information could be captured as part of a larger monthly staff report form, such as illustrated in Appendix. Alternatively, there are web-based faculty practice modules available from certain vendors that may be an appropriate solution for larger clinics. One example of such commercial solutions is offered by Typhon Group (http://www.typhongroup.com/) which markets software management solutions for allied health training and residency programs. Typhon also markets a “Faculty Practice” module for the administration of the clinical activities associated with allied health clinical staff. Examples of activity reports from the Faculty Practice module for a patient encounter are given in Tables 2–4. Of course, these tables are relatively simple and could also be easily reported using a spreadsheet software application. Tables 5 and 6 show how competency reports for individuals and clinical team members might be reported.

**Table 2:** **This demographic information needed to report a staff encounter with a patient service is illustrated**.

Case ID: 123-456-7890	Date of service: 2013 February 2
Faculty/staff information	Rosalyn Blue
Semester/quarter	Winter, 2013
Course	Clinical special procedures
Clinical faculty	Albert Einstein, chief of physics
Clinical site	State University Cancer Center, radiation oncology

**Table 3:** **Clinical information of a staff/patient encounter is listed**.

**CLINICAL INFORMATION – ROSALYN BLUE**	
Time with patient	180 min
Consult with other clinical faculty	60 min
Faculty participation	Complex skills used
High dose rate brachytherapy	Plan breast (not MammoSite) (done)
High dose rate brachytherapy	Plan GYN (not T&O) (done)

*It is indicated whether the encounter is observed, assisted, or done. Those cases performed under supervision count for training/maintenance of competency*.

**Table 4:** **These conference logs demonstrate the process to evaluate the partial requirements for a staff medical physicist to maintain clinical competency through educational activities**.

Date	Topic/speaker	h	CME/CEU	CRM?
**CONFERENCE LOGS – ROSALYN BLUE**				
1/21/2013	HDR T&O/Dr. M. Curie	2	N	No/N/A
2/5/2013	Peer review/Dr. F. Radical	2	N	No/N/A
2/10/2013	Conducted reviewer peer review of medical physics article: treatment planning quality assurance for Gamma Knife treatment delivery	2	N	No/NA
2/16/2013	CyberKnife lung SBRT/Dr. S. Cooper	2	N	No/N/A
3/1/2013	Total skin irradiation/Dr. G. Khan	2	N	Y/Y
3/5/2013	Trained physics resident J. Newby in TBI setup	2	N	Y/Y
3/15/2013	TomoTherapy IGRT/Dr. R. Balboa	2	N	Y/Y
3/20/2013	Presented JACMP article in journal club: quality assurance for helical TomoTherapy SBRT lung planning	2	N	Y/Y
3/27/2013	Iodine 131 ablation/Dr. N. Reactor	2	N	Y/Y

*CRM, competency requirement maintained*.

**Table 5:** **This example competency report demonstrates the process to evaluate the partial requirements for a staff medical physicist to maintain clinical competency through clinical service**.

Observed	Assisted	Done	Min. required	Item (category)	Competency assigned?	CRM?
**COMPETENCY REPORT – ROSALYN BLUE**						
0	1	3	2/quarter	Total skin irradiation	Y	Y
0	0	2	2/quarter	Total body irradiation	Y	Y
0	1	5	5/quarter	Stereotactic body radiotherapy	Y	Y
0	1	5	5/quarter	Stereotactic radiosurgery	Y	Y
0	0	4	2/quarter	Intraoperative radiotherapy	Y	Y
0	0	0	2/quarter	Low dose rate brachytherapy	N	N
0	0	0	2/quarter	Prostate seed brachytherapy	N	N
0	1	0	3/quarter	High dose rate brachytherapy	N	N
0	1	3	2/quarter	Therapeutic nuclear medicine	Y	Y
0	0	8	2/quarter	CT/PET/MRI fusion	Y	Y
0	0	6	3/quarter	4D respiratory gating	Y	Y
0	0	0	5/quarter	Gamma Knife radiosurgery	N	N
0	0	0	5/quarter	CyberKnife radiosurgery	N	N
0	0	10	3/quarter	TomoTherapy IGRT	Y	Y

*CRM, competency requirement maintained*.

**Table 6:** **The physics clinical staff competency spreadsheet can be set up to tabulate the data entry from Tables 4 and 5 and demonstrate the ongoing fulfillment of local competency requirements**.

First quarter 2013; special procedure; physics staff competency record	Physics clinical staff								
	R. Blue			P. Walnut			M. Redfield		
	CR	CL	CRM?	CR	CL	CRM?	CR	CL	CRM?
Total skin irradiation	Y	Y	Y	Y	Y	Y	N	N	N/A
Total body irradiation	Y	Y	Y	Y	Y	Y	N	N	N/A
Stereotactic body radiotherapy	Y	Y	Y	N	N	N/A	Y	Y	Y
Stereotactic radiosurgery	Y	Y	Y	N	N	N/A	Y	Y	Y
Intraoperative radiotherapy	Y	Y	Y	N	N	N/A	Y	Y	Y
Low dose rate brachytherapy	N	N	N/A	Y	Y	Y	Y	Y	Y
Prostate seed brachytherapy	N	N	N/A	Y	Y	Y	Y	Y	Y
High dose rate brachytherapy	N	N	N/A	Y	Y	Y	Y	Y	Y
Therapeutic nuclear medicine	Y	Y	Y	Y	Y	Y	Y	Y	Y
CT/PET/MRI fusion	Y	Y	Y	Y	Y	Y	Y	Y	Y
4D respiratory gating	Y	Y	Y	Y	Y	Y	N	N	N/A
Gamma Knife radiosurgery	N	N	N/A	N	N	N/A	Y	Y	Y
CyberKnife radiosurgery	N	N	N/A	Y	Y	Y	N	N	N/A
TomoTherapy IGRT	Y	Y	Y	Y	Y	Y	N	N	N/A

*CR, competency report table; CL, clinical logs table; and CRM, clinical requirement maintained*.

## Discussion and Conclusion

Although this formalism is relatively simple, only a vanishingly small number of radiation oncology centers address the issue of continued local credentialing and special procedures practice validation. There are probably several reasons for this. If the center employs only one or two medical physicists, these are already obligated to make their best effort to maintain competence in all technologies offered. Designing and maintaining a local credentialing spreadsheet is likely beyond the technical awareness and knowledge of radiation oncology administrators and administrative vice-presidents that constitute the normal reporting chain in the smaller centers. For the medical physicists, if no one is checking up on our competency and asking for documentation, why bother to do it? In general, is that not what maintenance of certification is designed to accomplish?

For larger centers employing 10 or more medical physicists, the amount of recording and oversight can escalate to an unmanageable level. Again why go to the trouble if evidence of competency is what maintenance of certification is intended to accomplish? Why do this if it does not help patients to be treated better in some measurable way? Should local credentialing activities prove their cost-effectiveness just as other patient safety programs are expected to do? Finally, since local credentialing is mostly not a part of the radiation oncology culture, is there sufficient energy around the broad inertia of “patient safety” to accomplish this significant culture change?

Possibly the answer is that local credentialing is simply the right thing to do. It adds a measure of safety to radiation oncology interventions that are already characterized by accuracy, precision, and some risk. Also, it provides some measure of protection to the chief of physics, the administration, and the institution if at some point difficult questions need to be answered following a medical event.

Finally, this formalism is arguably too simplistic. Some would say it cannot really accomplish the goal of local credentialing because it fails to measure competency in sufficient detail. Each medical physics director will decide what level of detail is needed locally. A minimal program likely is better than no program at all, and the perfect is often the enemy of the good. This approach is therefore respectfully offered as an additional step toward making radiation oncology deliveries safer and more consistent in the broader community.

## Conflict of Interest Statement

The author declares that the research was conducted in the absence of any commercial or financial relationships that could be construed as a potential conflict of interest.

## Appendix

### Sample monthly physics report

**Physics consultation report**

**Physicist:** choose an item. **Month:** choose an item. **Date:** click here to enter a date.

**Table:**

Week	Number of charts	Hours checking	Clinical coverage hours
Week 1	Choose an item	Choose an item	Choose an item
Week 2	Choose an item	Choose an item	Choose an item
Week 3	Choose an item	Choose an item	Choose an item
Week 4	Choose an item	Choose an item	Choose an item
Week 5	Choose an item	Choose an item	Choose an item

Special medical physics consultation – all fractions for one patient.

**Table:**

Procedure	Number performed	Abt/local report hours	Total hours
Total skin irradiation	Choose an item	2.0	Choose an item
Total body irradiation lateral	Choose an item	2.0	Choose an item
TBI Mick frame	Choose an item	9.0	Choose an item
High dose rate brachy	Choose an item	4.0	Choose an item
HDR special procedure	Choose an item	Choose an item	Choose an item
Stereotactic body XRT	Choose an item	11.0	Choose an item
Stereotactic radiosurgery	Choose an item	7.0	Choose an item
Stereotactic radiotherapy	Choose an item	11.0	Choose an item
Intraoperative XRT	Choose an item	4.0	Choose an item
Prostate seed brachytherapy	Choose an item	4.0	Choose an item
IMRT (plan and review)	Choose an item	6.0	Choose an item
IMRT (plan review only)	Choose an item	2.0	Choose an item
IGRT (guidance only)	Choose an item	1.0	Choose an item
LDR intracavitary/interstitial	Choose an item	2.0	Choose an item
Therapeutic nuclear medicine	Choose an item	2.0	Choose an item
Other 77370 (e.g., fusion)	Choose an item	2.0	Choose an item

I have a current research project: □ If so, name research project

**Work on articles for publication**

**Hours worked:** choose an item. **Number accepted:** choose an item. **Number published:** choose an item.

**Work on posters or presentations**

**Hours worked:** choose an item. **Number accepted:** choose an item. **Number presented:** choose an item.

**Work on grants**

**Hours worked on grants:** choose an item. **# Of grants approved:** choose an item. **Amount:** $ click here to enter text.

**Mentoring hours for physics residents**

**First year resident:** choose an item. **Second year resident:** choose an item. **Physics assistant:** choose an item.

**Educational program hours for radiation oncology residents and physics residents**

**Rad. Onc. residency:** choose an item. **Therapy physics residency:** choose an item. **Cont. Ed.:** choose an item.

**Service hours**

**UL committee:** choose an item. **National organizations:** choose an item. **US government:** choose an item.

**Monthly QA, annual calibrations, special projects: commissioning, RPC projects, clinic projects, radiation safety**

**Describe project:** click here to enter text. **Hours worked:** choose an item.

**Time away (hours)**

**Administrative leave:** choose an item. **Vacation:** choose an item. **Sick:** choose an item.
